# We can evaluate rapidly, but should we? Researchers’ and research funders’ perspectives on the uses, challenges and limitations of rapid health care evaluation

**DOI:** 10.1177/13558196251340549

**Published:** 2025-05-07

**Authors:** Jo Ellins, Kelly Daniel, Manbinder Sidhu

**Affiliations:** 1 Senior Fellow, Health Services Management Centre, 1724 University of Birmingham, Birmingham, UK; 2 Evaluation Fellow, Health Services Management Centre, 1724 University of Birmingham, Birmingham, UK; 3 Associate Professor, Health Services Management Centre, 1724 University of Birmingham, Birmingham, UK

**Keywords:** evaluation, rapidity, quality

## Abstract

**Objectives:**

There is increasing demand for rapid evaluation in health care to inform timely policy and practice decision-making. This qualitative study explored the perceived benefits, limitations and challenges of rapid evaluation, focusing on how considerations of timescale and research quality are balanced in study design and delivery in England.

**Methods:**

We conducted fifteen semi-structured interviews with researchers and research funders involved in rapid evaluation, based in England. Data were thematically analysed using the Framework Method.

**Results:**

Results are reported around five major themes: (i) rapid evaluations are purpose driven; (ii) ‘good enough’ evidence; (iii) trade-offs and limitations; (iv) mitigating the speed and rigour trade-off; and (v) deciding if and when to evaluate rapidly. Study participants agreed that rapid evaluation reflected a drive to better align evaluative processes and outcomes to the needs of service planners and policymakers. It was seen to generate quick data for short-term requirements, and information to justify the need for, and inform the design of, longer-term assessments. However, working rapidly could restrict or prohibit some research activities, and there were particular concerns about recruitment being limited to sites and participants that were easier to access in short timescales. Rapid evaluation was considered less suitable for ‘high stakes’ topics or decisions, where evidence robustness and generalisability was paramount. Several study participants had built an infrastructure to facilitate rapid working which, at least in part, reduced the need to make methodological compromises.

**Conclusions:**

Rapid evaluation can support real-time learning for innovation and improvement and inform time-critical decisions, but timeliness is only one factor in the production of useful and usable evidence. It is a tool for specific circumstances and purposes, to be used alongside, rather than instead of, long-term and longitudinal designs.

## Introduction

Evaluation has an essential role to play in health care innovation and improvement.^1^ Evaluation studies seek to provide insights into whether and, increasingly, how policies, services or interventions are working. They also generate and catalyse formative learning, which can support implementation, adaptation, spread and scale-up processes. The potential for health care evaluation to inform decision-making is well recognised,^2^ but so are the various factors that can limit end-users’ engagement with evaluation findings.^3,4^

Timeliness is one such factor. Insights from evaluations must be available within an ‘actionable’ timeframe if they are to inform decision-making and improve care,^5^ and this is ever more relevant in light of an increasingly rapid pace of health care policy making and innovation. There have been calls for realignment of the timescales for research and evaluation activity with those of policy and practice,^6,7^ with Duncan and Harrop noting that “*policy makers’ and practitioners’ decisions could not wait for the cast‐iron answer, even if this were possible. Decisions are made on the basis of available evidence, be this imperfect or not”*.^8(p. 167)^

There is increasing demand for rapid evaluation in health care.^9,10^ While calls for rapid evaluations are not new,^11^ they have intensified in recent years with the unprecedented acceleration of research commissioning and delivery timescales in response to the COVID-19 pandemic.^12^

### Defining rapid evaluation

There is no consensus about what constitutes ‘rapid evaluation’. It tends to be understood in terms of timing and approach, with timing referring to a relatively short overall timescale that is more directly aligned with decision-making processes or windows. Views of what counts as a rapid timescale vary and this can range from durations of six days to 3 years.^10^ Rapidity might also refer to contracted time periods for commissioning or mobilising a new study, or for reporting findings.^5^ Some studies involve phases of rapid data collection and/or analysis within a longer-term duration. This is also known as rapid cycle evaluation, further blurring the demarcation between short- and long-term designs.

Rapid evaluation is also used to describe a range of approaches that can deliver expedited results. Norman et al.^9^ describe four main types: (i) using a methodology specifically designed for rapid evaluation; (ii) reducing the scope or extent of data collection, or using a less time-intensive methodology; (iii) using alternative technologies to rapidly acquire and/or analyse data, or using existing data sets; and (iv) designing discrete elements of non-rapid studies that are undertaken rapidly. In qualitative research, approaches include analyses based on recordings or notes, eliminating the need for transcription, and methods for rapidly summarising data, such as mind maps or structured rapid assessment procedure (RAP) sheets. In quantitative research, scenario-based counterfactuals, measurement of interim endpoints and techniques to model longer-term outcomes from early data have been reported.^5^

### Balancing rapidity and rigour

While health care decision-makers want quicker evaluation findings, and researchers have at their disposal a growing array of methods for delivering these, there are nonetheless reasons for being cautious about the current trend towards more rapid evaluation. A key concern relates to the balance of rapidity and rigour. Working to tight deadlines may require strategies such as reduced sample sizes, shorter fieldwork, and simplified or more rapid data analysis.^9^ Short timescales can also limit the time available for assessing and assuring the credibility of results, such as data triangulation and member-checking.^13^ For these reasons, rapid evaluation has, on occasion, been described as being ‘quick and dirty’.^14^

Not all researchers agree that these problems are inherent to rapid evaluation, and there are very few studies that have directly compared rapid and non-rapid approaches, which could help to resolve this matter.^9^ One study analysed the same qualitative dataset using rapid and non-rapid analysis approaches, and found considerable overlap in the results and recommendations; however, the use of a more deductive approach to achieve rapidity generated less depth and detail.^15^ It has been argued that much of the concern around rapid evaluation is driven by its pragmatic and practice-oriented nature, rather than rapidity as such.^16^ Further, many sources of bias and poor quality are unrelated to timescale.^17^ Rather than being seen as in tension, rigour and rapidity could instead be regarded as two equally important aspects of methodological quality, which must be kept in balance, with McNall and colleagues arguing that “[t]he timeliness of this information is no less critical than its accuracy”.^18^ ^(p. 287)^ Efforts to define and ensure rigour in this field of research, including a planning and reporting framework for rapid qualitative analysis and the development of international standards for rapid evaluation and appraisal methods are underway.^19,20^

This study aimed to explore the benefits, limitations and challenges of rapid evaluation in health care from the perspectives of those who are engaged in this type of work, either as researchers or research funders. It specifically focused on considerations of timescale and quality and how these are balanced in practice as rapid evaluation studies are designed and delivered. We further sought to understand what considerations shape assessments about whether to proceed with a rapid evaluation and draw insights about the place and contribution of rapid evaluation in the wider applied health services research landscape.

## Methods

We conducted a qualitative empirical study involving semi-structured interviews with people directly involved in the commissioning of and/or carrying out rapid evaluation in health care.

### Participant sampling and recruitment

Study participants were identified through: (i) the list of attendees of a national rapid evaluation in health care conference held in London, England in January 2021; (ii) a database of studies funded by the United Kingdom Research and Innovation (UKRI) COVID-19 Rapid Response funding scheme; and (iii) web searches to identify research teams or organisations in England specialising in rapid health care evaluation. Information on potential participants was gathered through websites and other public sources, and priority was given to individuals for whom there was evidence that they were actively involved in rapid evaluation (e.g. researchers whose web profiles included details of rapid evaluation studies they were leading or involved in). We specifically sought to identify individuals involved in health *services* research and excluded people who involved, mainly, in clinical or epidemiological research.

### Data collection

Participants were approached by email with information about the study and asked to complete and return a consent form if they were willing to take part. Eleven people responded to our invitation; four additional participants were identified through recommendations made in the initial interviews. Semi-structured interviews followed a topic guide that was informed by the study aims and objectives, and a preliminary review of relevant empirical and methodological literature. All interviews were undertaken between September and November 2021, and conducted remotely using the Zoom video platform. Interviews lasted between 25 and 57 minutes; they were digitally recorded following consent and transcribed verbatim. Transcripts were checked for accuracy, with researchers returning to the original voice recording to address any transcription gaps or errors.

### Data analysis

Data were analysed thematically using the Framework Method.^21^ This is a structured process for qualitative analysis specifically developed for applied and policy-relevant research; it involves familiarisation, identification of a preliminary thematic framework, coding, charting and interpretation. Two researchers (JE and KD) carried out the interviews, and each initially familiarised themselves with the data from the interviews they had conducted. A preliminary coding framework was developed based on initial reading of the interview transcripts, the study aims and literature review. This was then applied to the data, with further codes identified inductively from the analysis. Each researcher independently reviewed a sample of the other researcher’s transcripts to ensure consistency in the application of the framework. A structured template was developed for each researcher to summarise their data to support comparative analysis and data synthesis. The researchers met regularly during the analysis period, including three data analysis workshops to discuss identified themes, explore commonalities and differences between participants’ responses, and incorporate insights from the wider literature.

### Ethical approval

This study was approved by the University of Birmingham Humanities and Social Sciences Research Ethics Committee (ERN_21-1229).

## Results

Of a total of 20 people invited, fifteen agreed to take part in the study. The final sample included eleven participants who identified as ‘researchers’ and four participants who identified as ‘research funders’. All except one participant worked in organisations based in England, including public bodies or public interest organisations (*n* = 6), universities (*n* = 5), research consultancies (*n* = 2) and charities (*n* = 2). One participant had recently moved to another country, but the interview focused on their many years of experience of working in research in England.

We report the results for five major themes identified in the analysis: (i) rapid evaluations are purpose driven; (ii) ‘good enough’ evidence; (iii) trade-offs and limitations; (iv) mitigating the speed and rigour trade-off; and (v) deciding if and when to evaluate rapidly.

### Rapid evaluations are purpose driven

There was consensus among interview participants that rapid evaluation produced learning and evidence for specific, practical and time-bound purposes. The primary driver for an evaluation to be undertaken rapidly was an evidence need within a tightly constrained timeframe. This was because action had to be taken for a fixed deadline determined by external factors (e.g. political or funding cycles), or because the evolving nature of the intervention or the context for its implementation meant that a delay in the findings could render them out of date.

There’s a sense of urgency. So there’s a kind of policy or practice uncertainty, where either the service is developing quickly, so there’s an opportunity, there’s a window for assessment which can then inform further roll out or development. But it’s, there isn’t the luxury of the kind of three to five year window of a formal sort of funded research evaluation. [Participant 2, research funder]

The rising interest in rapid evaluation was seen as a significant and largely positive development, reflecting a drive to better align evaluative processes and outcomes to the needs of service planners and policymakers. Participants frequently highlighted the potential for timely evidence to inform iteration, adaption and course correction as interventions were tested in real-world settings as a major motivator for conducting rapid evaluation.

I think the benefit is that you’re able to course correct…You’re evaluating a new service or something like that, so being able to sort of course correct rather than have to sit back for three years and see whether it worked or not. I think the [National Health Service] is under too much pressure to take that approach at the moment. [Participant 14, researcher]

One study participant noted that rapid evaluation was often described as being demand-driven but, in their experience, evidence users and researchers shared the same goal for more responsive and usable findings.

I think it’s at both ends of the, you know, down in the reeds and up in the ivory tower there is dissatisfaction, isn't there. And also dissatisfaction from people who put money into those ends, either of those ends, that too much stuff on the ground blunders on without ever asking ourselves, do we make enough of a difference here? [Participant 12, research funder]

At the same time, there was some concern about the logic underlying the purpose-oriented case for rapid evaluation, in particular as it relates to an assumed causal relationship between the timeliness of evidence and its uptake.

It’s tied up with the idea that research and evaluation should have a fairly direct and linear relationship to policy and management decisions, and that if a so-called novel evaluation doesn’t have such a direct relationship, this is in part, often in large part, because it isn’t quick enough. So this is the idea that if we could do things more quickly then [findings] would be more useful and therefore they would be more likely to be used. I think there’s quite a lot of big assumptions underlying that. [Participant 8, researcher]

Several interview participants commented that stakeholders did not always engage with or use evaluation findings, even when these were produced within an agreed rapid timescale and where there was strong stakeholder input into shaping the evaluation scope, questions, methods etc. Many said that there was a need for better understanding of how to optimise evaluation evidence for policy and practice use and, conversely, how to make planning and decision-making processes in policy and practice more receptive to the use of evidence.

I think you're going to have much more impact on [the research] side if you also kind of think about the other side. Like, what would a good consumer of good evaluation look like, what questions would they ask themselves and maybe do organisations need a little bit of help with that. [Participant 12, research funder]

A small number of study participants noted that the increasing use of rapid approaches was also driven by funding constraints. Shortening data collection and/or analysis processes may mean that studies can be undertaken at lower cost, and this might be the only option available when the budget was small.

Other pressures are the pressures that you feel from the team side, where you don’t have large budgets and where sometimes you need to focus on rapid designs because you have very limited funds… [Evaluation teams] have to be quite creative with their budgets. [Participant 14, researcher]

### ‘Good enough’ evidence

A recurring theme was that rapid evaluation addressed real-world evidence needs, that it aimed to produce the kind of information that people making decisions ‘on the ground’ actually needed and could use. Study participants were, however, aware that some in the research community considered rapid evaluation to be of poorer quality compared to long-form studies. Some participants related this to existing frameworks for understanding and assessing evidence quality, which were seen to be overly focused on study design and methodological rigour, with insufficient attention paid to other dimensions of value such as timeliness and relevance.

What evidence-based medicine has done is it’s created this decontextualised set of criteria and assessment framework, when actually surely what is the best kind of evidence is the evidence that’s there when you need it because if you get the answer four years later…The world’s moved on! [Participant 4, researcher]

In contrast to the evidence-based language of ‘gold standard’ research, rapid evaluations were frequently described as producing ‘good enough’ evidence, meaning that they generated evidence that was fit for the purpose for which it was required. There was a suggestion that rapid evaluations were designed with the twin considerations of evidence quality *and* adequacy in mind.

I also thought about this idea of research being good enough for the needs of the customer…[In contrast to] a rather elitist view of life that only a randomised control trial which is double blind, blinded is worth the money, because we all know that’s not the case, not least because that doesn’t answer all the questions that people have. [Participant 5, research funder]

Study participants recognised the need for trade-offs to be made to deliver a study within a tight timeframe, but these could be justified where the choice was between decision-makers having some (albeit imperfect) information or having none at all.

It’s about shifting standards and making compromises to meet the speed requirement, because there is an argument that getting some data quickly is better than coming up with the perfect answer by the time everyone’s made a decision and moved on. [Participant 9, researcher]

One participant noted that evaluation research was inherently pragmatic in nature and that choices and value judgements had to be made to achieve a balance between scope, rigour and depth, within available resources. There was recognition that rapid evaluations may not be able to answer all questions, but that other forms of evaluation would unlikely offer a definitive answer or complete certainty either. Rather, like all forms of knowledge production, evaluation research was seen to proceed incrementally and iteratively.

There was agreement that rapid evaluation could generate quick data for short-term requirements and also provide information to justify the need for, and inform the design of, more detailed and longer-term assessments.

So you could see almost a continuum or certainly something that’s got some sense of phasing. So a rapid evaluation might make the initial decisions, but it might also have to design the evaluation strategy for the rollout of the bigger programme or the practise or whatever it’s going to be. [Participant 1, researcher]

There were mixed views on whether the term ‘rapid evaluation’ was helpful, given that the methodological considerations and challenges of rapid and non-rapid studies were not fundamentally different. One study participant suggested that the term ‘proportionate evaluation’ might better capture the core work involved in aligning evaluation purpose, design and timescale, and avoid connotations of being ‘slapdash’. Others felt that the term rapid evaluation should continue to be used to describe studies that had been carried out at an accelerated pace for a specific purpose.

### Trade-offs and limitations

The need for trade-offs was a common and accepted feature of rapid evaluations, but not always something that researchers felt comfortable with. Participants noted the difference between acceptable and unacceptable trade-offs. Unacceptable trade-offs could fundamentally compromise the integrity of the research or deviate from recognised ethical standards.

There are certain basic principles that apply to any type of research which have to be adhered to. It’s about probity, it’s about integrity, it’s about ensuring that the right guidelines and frameworks are adhered to. [Participant 5, research funder]

Participants distinguished three broad types of trade-off. The first concerned research activities that were de facto precluded, particularly where ‘rapid’ meant a short overall timescale, such as tracking of implementation processes or outcomes over time and measuring outcomes that would likely not occur within the evaluation timeline.

So you know if your principal objective with the evaluation is to explore the impact of an intervention on a set of outcomes then clearly there’s a rate limiting factor, which is the outcome. You can’t report on something that hasn’t changed…Because it hasn’t had time to change. [Participant 11, researcher]

Some participants suggested that rapid approaches were therefore most appropriate for early and formative assessments, and less well suited to summative evaluation. It was also noted, often with evident frustration, that tight timescales could also prohibit some forms of primary data collection, especially where this involved patients, given the time needed to secure necessary ethical and governance approvals.

In one rapid evaluation we did, we wanted to speak to some patients in a mental health setting. As evaluators we knew that was something we needed to consider very carefully, from an ethical standpoint, and take through the various routes. Those mechanisms were not quick enough to assure us of the approval that we needed to go into that setting, so we didn’t. [Participant 13, researcher]

Similarly, securing access to national datasets such as hospital utilisation data was often not possible within short evaluation timeframes. A second type of trade-off described by interview participants related to trading rapidity against study scope to preserve quality. This meant data collection and analysis were undertaken with a high level of rigour but the evaluation would address a more narrowly defined focus and set of research questions. A third type related to the ways in which methods had been designed to enable delivery within a rapid timescale. Examples included: conducting more ‘light touch’ analysis and synthesis of data using rapid techniques; using convenience or snowball samples, rather than representative or purposive approaches; collecting data using online platforms and tools, or drawing on an established infrastructure (e.g. adding questions to an existing panel survey); foregoing respondent checking or validation; and collecting large amounts of data using large-group workshops, crowdsourcing methods or social media. Some study participants highlighted the creativity and innovation in research methods that rapid evaluation was felt to be driving.

In terms of limitations, the main one discussed by participants was the risk that, if the time available for recruitment was highly constrained, study samples would be limited to better performing areas, which were generally more willing to take part in research, and to participants that researchers found easier to identify and access. This could further marginalise groups that were already poorly represented in evaluation research, and hamper efforts to assess inequalities in access, experience and outcome. Participants also felt that there was a risk that studies focused on variables that were easiest to measure or for which researchers could readily access existing data, which might not necessarily be those that were most important or relevant.

Particularly when there’s a certain amount of urgency in rapid evaluation there’s the risk to look at the metrics that we already have access to…A lot of what we do is look at hospital activity as an outcome, which isn’t necessarily always the best metric to judge an intervention against. [Participant 14, researcher]

It was noted that the trade-offs and limitations that might need to be made in rapid evaluation could lead to more uncertain or ambiguous findings, which were more difficult to communicate to evidence users in a simple, clear and understandable way.

But to communicate clearly the kind of results which might be interesting and so on while, you know, being suitably cautious based on the weight that the study can support, that’s really tricky. [Participant 2, research funder]

### Mitigating the speed and rigour trade-off

Several participants described how they had built an infrastructure to facilitate rapid working which had, at least in part, reduced the need for methodological compromises. What came through in these comments was that the capability to work rapidly had often been carefully planned and constructed in advance. Identified factors included having the ‘right’ team, in terms of both researcher skills and mindset. Studies could be mobilised and delivered more effectively in short timescales with researchers who were experienced in conducting rapid evaluations and the techniques and approaches involved, and willing and able to work in a fast-paced, responsive and pragmatic way.

I think probably something about the mind-set of the staff team that this is, you know, the need to adapt, the need to kind of always be flexible to the stakeholders needs and fit accordingly rather than necessarily doing the same method every time. [Participant 7, research funder]

The ability to quickly access relevant topic area knowledge and expertise to support study design processes, and data collection, analysis and interpretation was also seen to be important. One study participant described how their team had developed the skills to rapidly convene expert advisory panels, and that these had been crucial for successfully delivering short-term studies. Other factors included having existing tools and processes in place that could be quickly adapted for new studies, such as research tools, analytical methods, project management techniques, stakeholder engagement processes, and standing advisory bodies. A small number of study participants worked in teams that had negotiated long-term access to national datasets or expedited ethical review by their organisation for certain types of service evaluation or in particular circumstances.

### Deciding if, and when, to evaluate rapidly

There was agreement that rapid evaluation was not suitable for all research questions and situations. Some participants noted that rapid evaluation should be used in situations where there was an urgent evidence need, which legitimised making methodological compromises to produce expedited results. Being judicious about when to use rapid evaluation was also felt to be important because of the heavy burden it was seen to place on researchers and participants.

Sometimes there is an urgent policy need or practice need to get evidence very rapidly, but you should almost save that rapid facility for those occasions when it’s really needed. Because it’s stressful, it places burden on both the research team and the participants and it’s more high risk because you're having to make things happen at particular speed. And there’s really no need to do that if there isn't a need to do it, if that makes sense. (Participant 1, researcher)

The limitations of evaluating rapidly made it unsuitable for what could be termed ‘high stakes’ situations or decisions, where the robustness and generalisability of the evidence was paramount. Several participants commented that they would be particularly cautious about undertaking a rapid evaluation to answer questions of patient safety. One participant noted that findings from rapid studies could be used in decisions about whether to continue funding a service or programme, but that such decisions ought only to be taken in conjunction with other sources of data and intelligence.

Deciding whether to proceed with a rapid evaluation was seen to involve balancing considerations of the type of evidence needed and how it was intended to be used, time and budget available, and nature and extent of methodological compromises that were anticipated. It was felt to be important that this process was undertaken in conjunction with relevant stakeholders, not only because they held information (e.g. about evidence need) vital to the process, but also because these were not purely technical decisions, they involved value judgements.

Let’s start at the other end of the spectrum and say ‘OK, who needs this work and what is it they need from this work and what would they find acceptable in terms of a level of whatever you want, evidence, proof, robustness, that would help them to be able to make the decisions that they need to take around whatever it is that we’re investigating? [Participant 6, researcher]

Several participants commented that stakeholders were not always clear, or in agreement, about what they wanted from a rapid evaluation. In such situations they sought to engage and foster dialogue between key groups in the goal of developing a shared understanding of information need and purpose. This initial work to understand stakeholder requirements, define study scope and assess feasibility was seen to be crucial but often complex, and some participants called for better guidance and tools to help researchers undertake these preliminary activities more effectively.

There was agreement that evaluation researchers needed to be clear and open with stakeholders about the anticipated limitations of the data that a rapid study would produce. This was seen to be important for managing expectations and, more generally, preventing evaluation commissioners and users reaching the mistaken view that all studies could, and should, be delivered rapidly.

Transparency is really important. So I think making sure that we still state and keep reinforcing those limitations to this work. Don’t get to a place where people say well that’s OK then, rapid evaluation is delivering us findings within three months, that’s fine, we don’t need anything else. Because that won’t be OK. [Participant 13, researcher]

Relatedly, some participants felt that there was often a rush to generate data for policy and practice through evaluation, when synthesising existing evidence or other methods of quickly generating insights to support decision-making (such as rapid consensus building approaches) might be more useful and feasible in the time available.

There are other ways of taking evidence informed decisions without necessarily expecting evaluators to work to extremely tight timescales. [Participant 8, researcher]

## Discussion

Our study examined the value of rapid evaluation as a means for providing more timely evidence for policy and practice. Despite important caveats, study participants were generally positive about the growing acceptability and use of rapid designs and approaches. Enhancing the usefulness of evaluation data for decision-makers was seen to be a key driver and incentive, achieved not only through rapidity but, as was often also described, ongoing stakeholder engagement in processes of study scoping, design and delivery. In this sense, rapid evaluation can be seen to reflect and advance wider debates about what counts as ‘best evidence’ in applied health research and related calls to recognise evidence usability as a core dimension of quality.^4,22^ Within time constraints, and particularly in conditions of urgency, idealised templates for evidence-based policy and practice must be set aside, and instead the guiding question for evidence makers and users becomes ‘what can be done’ in the time available.^23^

Questions remain about what constitutes ‘good enough’ data. Study participants described rapid evaluations as being fit-for-purpose, a view shared in the relevant methodological literature.^7,13,24^ This suggests that rapid evaluations must be understood and appraised in context, above all in relation to the specific evidence needs and intended uses that they are designed to address.^25^ Evaluation purpose is key for determining the level of detail and precision that is required and, therefore, when a rapid approach is suitable or sufficient, and the balance that should be struck between speed and rigour.^7^ Our findings showed that some questions or purposes may be too ‘high stakes’ for a rapid approach; assessing patient safety was the most frequently given example. This aligns with review evidence suggesting that rapid evaluations of health and social care innovations most often explore aspects of user experience and acceptability, and the barriers and facilitators of implementation in particular contexts, with only a small number of studies assessing issues such as vaccination safety in the context of the COVID-19 pandemic.^9^

Our findings highlight that a key task, and a central challenge, of rapid evaluation was deciding what trade-offs could be acceptably made. Participants described various ways of achieving rapidity, but there were also limits to how far speed could be traded off against rigour. Responding to a time-constrained or urgent need for evidence meant working quicky, but never outside of the ‘guide ropes’ of integrity and ethical practice. Being open with evidence users about what a short-term study could not realistically achieve was seen to be crucial, in order to avoid the risk that rapid approaches “could be over-sold, too rapidly adopted, badly done, and then discredited”.^26^ ^(p. 531)^

A major concern among participants in our study related to concerns around sampling, with implications for the generalisability and transferability of findings, and for efforts to increase inclusion and diversity in research.^27^ Effective involvement of minoritised and marginalised groups calls for tailored approaches, grounded in trust and relationship building, which focus on addressing barriers to participation.^28^ It is questionable whether the time and effort needed for this can be aligned with the demand for quicker evaluations, and this is a potential risk of rapid evaluation that requires careful attention.

Given the inherent and context-specific limitations of working at speed, our study highlights that rapid evaluation should be seen as a tool for use in particular circumstances, alongside, rather than instead of, long-term and longitudinal designs. Short-term studies provide only a snapshot at a point in time and may be tightly focused in scope, but they still contribute to the incremental advancement of knowledge, and can identify questions and evidence gaps that require more in-depth investigation.^5^ The constantly evolving nature of evaluative research suggests that distinctions, between ‘rapid’ and ‘non-rapid’, or ‘gold standard’ and ‘good enough’ evidence, are problematic and potentially misleading. Instead, as Nutley and colleagues propose, “it may be more helpful to think of an ‘evidence journey’ from promising early findings to substantive bodies of knowledge”.^25^ ^(p. 4)^

We note that the rationale and driver for increasing rapid evaluation is underpinned by an assumed causal relationship between the timing and use of evaluation results. Yet, as our findings confirm, while timeliness is an important consideration in maximising the value and benefit of evaluations, it is only one of many factors that influence whether and how evaluative evidence is used in policy and practice.^4^ These factors relate to the way in which evaluations are designed, conducted and communicated, and the extent of end user engagement in these processes; the receptiveness and skills of stakeholders to understand and engage with findings; and enablers and constraints in the wider organisational and political contexts in which evaluation takes place, including other influences on decision-making processes.^29,30^ As our study shows, rapidity may be necessary for ensuring that evaluations are useful and used, but it is rarely sufficient.

### Limitations

A key challenge of our study was identifying researchers and, in particular, research funders involved in rapid evaluation work. While we used several strategies to identify study participants, our sample was largely comprised of individuals working in public bodies and universities. It is possible that researchers and funders in other sectors, where organisational incentives and constraints may vary, could offer different insights into the place, value and implications of rapid evaluation. We were unable to explore whether there were such differences, although our sample did include a small number of participants working in charities and commercial organisations. Other designs, such as Delphi methods or focus groups, might have enabled more direct exploration of areas of consensus and difference, both across different sectors and between researchers and research funders. These methods were ruled out on feasibility grounds, due to anticipated challenges in identifying participants. The generalisability and transferability of the findings to other settings will need to be explored, as the research was carried out only in England.

## Conclusions

Rapid evaluation can generate real-time learning for innovation and improvement processes and inform time-critical decisions. There are inherent limitations to what can be achieved in a rapid timescale but those engaged in rapid evaluation had built an infrastructure to facilitate rapidity which reduced the need to make trade-offs in study design and delivery for the sake of speed. Our study suggests that there may be particular risks arising from shortening timescales for site and participant recruitment, which has the potential to exclude particular groups, experiences or outcomes that require more time to access and understand. While a rapid evaluation may be warranted in some circumstances, it may be less appropriate for ‘high stakes’ situations or decisions, where robust and generalisable evidence is paramount. Our findings point to the value of understanding rapid evaluation in a broader and longitudinal context, as a method that both contributes to the incremental advancement of knowledge, and which may precede and inform longer-term research.
